# Partial waivers: PEPFAR’s 2025 funding suspension and the looming HIV/AIDS catastrophe in Sub-Saharan Africa

**DOI:** 10.1186/s12977-025-00657-2

**Published:** 2025-03-31

**Authors:** Patrick Ashinze, Abba Inalegwu Owoicho, Michael Olanite

**Affiliations:** 1 Faculty of Clinical Sciences, University of Ilorin teaching Hospital, Ilorin, Nigeria; 2 Health Initiative for Safety and Stability in Africa, Abuja, Nigeria; 3 University Hospital, Dorset NHS Trust, Dorset, UK

**Keywords:** PEPFAR, HIV/AIDS, Expanded funding, Sub-Saharan Africa

## Abstract

Despite recent partial waivers granted by PEPFAR, the 2025 suspension of PEPFAR funding jeopardizes HIV/AIDS care for 20.6 million people, including 550,000 children, and risks reversing decades of progress in Sub-Saharan Africa (67% of global HIV burden). Immediate consequences include halted ART access, healthcare worker salary suspensions, and potential resurgence of AIDS-related deaths to 630,000 annually. Political disputes and funding misuse allegations further threaten program continuity. We urge expanded PEPFAR exemptions, rapid donor mobilization, and grassroots advocacy to avert catastrophe.

The Trump administration’s recent executive order halting nearly all United States foreign aid—including the President’s Emergency Plan for AIDS Relief (PEPFAR) [1, 2] —has precipitated a region-wide panic and is seeding a potential humanitarian crisis in Sub-Saharan Africa and other regions reliant on this lifesaving program. As clinicians, researchers, virologists, allied healthcare professionals and universal healthcare advocates committed to global health equity, we write to state the dire consequences of this decision and urge immediate action through united appeal to mitigate its fallout.

## The scope of the crisis

PEPFAR, established in 2003, has been a cornerstone of the global HIV/AIDS response, investing over 100 billion US dollars and providing antiretroviral therapy (ART) to over 20.6 million people annually, including 550,000 children, while supporting testing, prevention, and health infrastructure in 54 countries [2–4]. The program is credited with preventing an estimated 26 million AIDS-related deaths since its inception [4]. However, President Trump’s January 20, 2025, executive order mandating a 90-day review of foreign assistance has triggered an immediate suspension of PEPFAR’s operations, with no exemptions for global health programs [2, 3]. While partial waivers have since been granted for select activities, criticam components-including ART supply chains and healthcare worker salaries-remain suspended, leaving millions still at risk.

## Implications for Sub-saharan Africa

Sub-Saharan Africa, home to 67% of the global HIV burden, faces unparalleled risks. PEPFAR supports [5]:

- 222,000 daily ART collections; [5, 6]
- 224,000 daily HIV tests (diagnosing 4,374 people, including pregnant women) [5]
- 17,695 orphans and vulnerable children [5]
- 7,163 cervical cancer screenings [5]

The abrupt halt in funding disrupts drug supply chains, suspends salaries for 190,000 healthcare workers (many earning ≤$3,000/year), and risks treatment interruptions for millions [5, 6]. Even brief lapses in ART adherence can lead to viral rebound, drug resistance, grimmer morbidities and increased transmission—jeopardizing decades of progress [6]. 

### Political context and systemic vulnerabilities

The suspension reflects broader political tensions. Lawmakers have criticized PEPFAR following reports of $4,100 in funding being misused for abortions in Mozambique—a violation of U.S. law [6, 7]. While the incident was resolved, it has fueled skepticism about the program’s oversight, citing corruption in the rank and file of the healthcare systems in recipient countries and complicating international relations [7]. Concurrently, the administration frames the pause as a “moral imperative” to realign aid with U.S. interests, dismissing its role in pandemic prevention and global stability [7]. The world Health Organisation has expressed its deep concerns about the socioeconomic and sociodemographic implications [6], which stand odious— judging by The US’s controversial and recent exit from the grand organisation [8]. 

### Epidemiological outcomes

The World Health Organization warns that prolonged funding gaps could return global HIV mortality to 1980s levels, with 630,000 deaths annually [9]. Sub-Saharan Africa, where 62% of new infections occur among adolescent girls and young women, faces heightened vulnerability. Active and practicable resolutions are requisite to forestall a resurgence of unprintable calamities [10], as despite the reactant waiver policy, most PEPFAR programs aren't still receiving funding and are relatively inactive. [11].

### Recommendations

1. Expand PERFAR: Waivers: while partial exemptions exist, the US government must broaden waivers to cover all critical programs, ensuring uninterrupted ART access and healthcare worker support.
2. Emergency Funding from Alternative Donors: The Global Fund, EU, and philanthropic organizations should bridge gaps via rapid disbursements.
3. Leverage Local Stockpiles and Generic Production: Governments and NGOs must audit existing ARV stockpiles and collaborate with generic drug manufacturers (e.g., in India) to sustain supplies through indigenous production.
4. Strengthen Domestic Health Systems: Sub-Saharan nations must accelerate plans to integrate HIV care into national budgets, though this would require international debt relief, political will and technical support.
5. Advocacy: Grassroots campaigns, led by humanitarian groups and societies, can amplify patient voices and hold policy makers accountable.

## Conclusion

This suspension is not merely a policy shift—it is a malady for vulnerable populations. While partial waivers offer a hope and ease, they remain insufficient to prevent a resurgence of AIDS-related mortality. The global health community must act decisively to expand exemptions, secure funding and honor PEPFAR’s legacy. We call on every outlet of HIV? AIDS care, research and education to amplify this urgency, urging world leaders to prioritize humanity and empathy over every other salient reason or cause.

Shortly after the timing of the writing and acceptance for publication of this commentary, the US government offered a partial waiver to the cause of HIV/AIDS care worldwide. Yet still, concerned quarters are still at large underserved and in dire need of a full exemption. Our commentary notes this and staunchly advocates for a full waiver that will guarantee the costs of operation and servicing of human resources in the great grand battle against the spread of HIV/AIDS.

## Data Availability

No datasets were generated or analysed during the current study.
